# Effect of Initial Fe Content on Microstructure and Mechanical Properties of Recycled Al-7.0Si-Fe-Mn Alloys with Constant Mn/Fe Ratio

**DOI:** 10.3390/ma15041618

**Published:** 2022-02-21

**Authors:** Dongfu Song, Yiwang Jia, Qing Li, Yuliang Zhao, Weiwen Zhang

**Affiliations:** 1 Institute of New Materials, Guangdong Academy of Sciences, Guangzhou 510650, China; ywjia2018@163.com; 2 National Engineering Research Center of Near-Net-Shape Forming for Metallic Materials, South China University of Technology, Guangzhou 510641, China; 3 School of Liberal Arts Education, Guangzhou College of Technology and Business, Guangzhou 510850, China; barbaralq@126.com; 4 School of Mechanical Engineering, Dongguan University of Technology, Dongguan 523808, China; zhaoyl@dgut.edu.cn

**Keywords:** initial Fe content, gravity settlement, Fe-rich phase, plasticity

## Abstract

The effect of initial Fe content on the iron removal efficiency, morphology evolution of the Fe-rich phase and the mechanical properties of the recycled Al-7Si-*x*Fe-1.2*x*Mn alloy during melt holding was studied using an optical microscope (OM), scanning electron microscope (SEM) and tensile testing. The results show that with the increase of the initial Fe content, the residual Fe concentration of the alloys gradually increased, and the corresponding removal efficiency of Fe gradually was increased to 77.67%. The type of Fe-rich phase in the alloys changes from α-Al_15_(FeMn)_3_Si_2_ to a mixture of α-Al_15_(FeMn)_3_Si_2_ and β-Al_5_FeSi, and its morphological evolution is as follows: coarse Chinese-script + polygon → dense Chinese-script + polygon → polygonal + dense Chinese-script + plate-like. Furthermore, the morphology of the Fe-rich phase in the slag changes from a polygonal shape to an irregular shape with a two-layer structure. The formation and increase of the inner layer with high Mn-content in the irregular-shape phase is the main reason for the increasing residual Fe content. The plasticity of the alloy increases obviously with the increase of the initial Fe content, but the formation of the β-Al_5_FeSi with plate-like morphology in higher Fe-containing alloy may hinder further improvement of the plasticity.

## 1. Introduction

Al-Si alloy has been widely used in automobiles, motorcycles, electronic appliances, mobile phone communications and other fields due to its high specific strength, specific rigidity, good casting molding, corrosion resistance and low expansion coefficient [1]. With the increasing depletion of aluminum ore resources and increasing requirements of environmental protection, secondary aluminum has gradually become the main source of Al-Si cast aluminum alloys, because of the advantages of low energy consumption and low three-waste emissions. However, the inevitable impurity elements seriously reduce the comprehensive mechanical properties of the secondary aluminum [2].

Fe is one of the most harmful impurities in recycled Al-Si alloys, and the formed Fe-rich phase has a detrimental effect on the plasticity of the alloy [3]. In particular, the platelet-shaped β-Al_5_FeSi (β-Fe) phase not only severely splits the matrix, but also promotes forming of casting defects, such as porosity and shrinkage, and deteriorates the properties of the alloy [4]. Another type of Fe-rich phase, such as α-Al_8_Fe_2_Si and α-Al_15_(FeMn)_3_Si_2_ (α-Fe), has a compact morphology with granular or Chinese-script shape, which greatly reduces the fragmentation of the matrix and harmfulness [5].

In the past 30 years, many scholars have transformed the morphology of the Fe-rich phase by adding neutralizing elements, such as Mn, Cr, Be, B, RE, V, Co, Sr, Sc, and melt treatment, changing the cooling rate [6,7,8,9,10,11,12]. In particular, Mn neutralization is the most common method to minimize the harmfulness of β-Fe, namely, transforming the β-Fe with a monoclinic structure to an α-Fe with a body-centered cubic structure [13]. However, Mn neutralization is deeply influenced by the chemical composition, cooling rate, and melt treatment, resulting in uncertain Mn content under different conditions [12,13,14,15]. Especially for high Fe-containing recycled Al-Si alloy, the coarse Fe-rich phases with high melting point and density cannot be completely eliminated at the same time, which does not facilitate the improvement of plasticity [16,17,18]. 

However, primary α-Fe provides a good carrier for reducing the Fe content by physical methods, including gravity settlement, electromagnetic filtration, centrifugal separation, and ceramic filtration [19,20,21,22,23]. The processes of Fe removal included two steps, (i) the primary α-Fe formed by adding Mn combined with melt insulation, and (ii) then separation from the melt by gravity, electromagnetic force, centrifugal force and ceramic adsorption. Cao et al. [24] reported that the higher content of initial Mn and Fe, lower melt holding temperature and longer holding time were leading to higher Fe removal efficiency during the melt holding process. Lopes et al. [20] combined the gravity settlement and ceramic filtration to remove the primary α-Fe phases, and the Fe removal efficiency reached more than 80%. Flores et al. [25] suggested that the Fe removal efficiency increased with the increase of Mn content. The Fe removal efficiency was as high as ~80% when the Al-9.5Si-2.2Mn-1.6Fe alloy melt was held at 878 K for 65 min. Moreover, our previous research suggested that melt holding after adding Mn not only reduced the Fe content but also transformed the Fe-rich phases into fine and compact α-Fe, which significantly improved the plasticity of alloys [19,26]. However, the influence of Mn content and the melt holding process on the Fe removal efficiency and the mechanical properties of alloy has been reported in detail, but the effect of initial Fe content lacks systematic and in-depth research.

In this paper, the effect of initial Fe content on the microstructure and properties of high Fe-containing secondary Al-Si alloys was studied during melt holding. The evolution of the morphology, size and distribution of the Fe-rich phase was analyzed in detail, which is beneficial to optimize the properties of secondary Al-Si alloys.

## 2. Experimental Procedures

The test materials were prepared by industrial pure aluminum (99.5%) and several commercial master alloys, including Al-20Si, A1-5Fe and Al-10Mn. The Al-7Si-*x*Fe-1.2*x*Mn alloys, where *x* = 0.8 wt.%, 1.2 wt.%, 1.8 wt.% and 2.4 wt.%, were designed. The preparation process in detail is: about 2 kg of these raw materials were preheated at 500 °C for an hour and heated to 800 °C; after holding for 1 h, the melt was cooled to 720 °C with the furnace cooling (cooling rate is about 2.5–3.5 °C·min^−1^); subsequently, adding about 10 g of refining agent into the melt and slightly stirring the melt with a Ti bell; the melt was held for 30 min and cooled to 610–620 °C with the furnace cooling; after holding for 30 min, the melt was poured into a steel mold with a size of 30 mm × 200 mm × 150 mm and preheated at 250 °C.

The samples were taken at the same position at the bottom of the ingots and the chemical composition of the ingots was tested by Optical Emission Spectrometer (SPECTRO-MAX, SPECTRO Analytical Instruments, Kleve, Germany), and the slag samples were also cut by a hand saw. The metallographic samples were prepared by mechanical grinding with 240, 1000 and 2000 grit papers, polishing with 5–10% MgO aqueous solution, in turn, etching with 0.5% HF aqueous solution for 30–60 s and drying with cold flowing air. The microstructure observation and chemical composition were examined by an optical microscope (OM, Leica DMI 3000, Leica Microsystems, Wetzlar, Germany), and a scanning electron microscope (SEM, JEOL JXA-8100, JEOL, Tokyo, Japan) equipped with energy-dispersive spectrometry (EDS, OXFORD-7412, Oxford, UK). Based on more than five backscattered images of 200–500 times, the characteristic of the Fe-rich phase included area fraction, equivalent diameter, roundness was calculated by Image Pro-Plus 6.0 software. The tensile test bars were machined to “dog-bone” type and tested by a GP-TS2000A (Sinotest Equiment Co., Ltd., Changchun, China) at a tensile speed of 2 mm·min^−1^, the average of more than three valid values as the measurement. Based on the Pan Aluminum 2016 database, the equilibrium solidification phase diagram of Al-7.0Si-*x*Fe-1.2*x*Mn alloy was simulated and calculated by PANDAT^TM^.

## 3. Results and Analysis

### 3.1. The Effect of Fe Content on Fe Removal Efficiency

Table 1 presents the chemical composition and Fe removal efficiency of alloys with different initial Fe contents. After adding Mn and melt holding, the content of Fe and Mn in the alloys was significantly reduced compared to the initial contents, and the variation range of Si content was in the normal range. With the increase of the initial Fe content, the remaining Fe content in the alloy gradually increases, while the Mn content presented a trend of first increasing and then decreasing. The Fe removal efficiency gradually increased with the increase of initial Fe content and stabilized at 77.67%. In addition, the Mn/Fe ratio in the alloy also gradually decreased with the increase of initial Fe content.

### 3.2. The Effect of Fe Content on Microstructure

**Figure 1:**
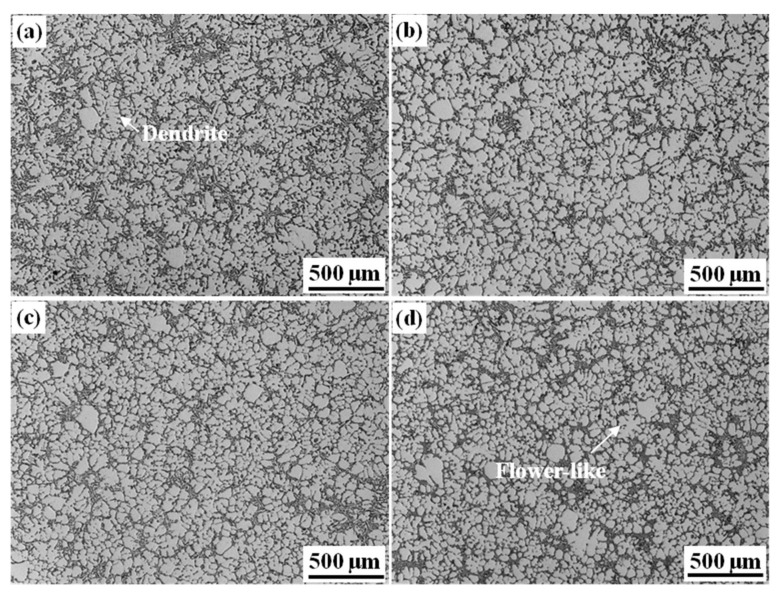
Optical images of alloys with different initial Fe content: (**a**) 0.8%; (**b**) 1.2%; (**c**) 1.8%; (**d**) 2.4%.

Figure 1 shows the optical images of ingots with different initial Fe content. The morphology of Fe08 alloy was mainly composed of α-Al dendrites, some petal-like and equiaxed grains; the macrosegregation of eutectic silicon was obviously distributed in the dendrite gap. With the increase of Fe content, the morphology of the α-Al dendrite was transferred to petal-like and equiaxed, and the coarse dendrites nearly disappeared, additionally, the segregation of eutectic silicon decreased. In addition, when the initial Fe content exceeded 1.2%, the morphology of α-Al dendrite of alloys did not change significantly.

**Figure 2:**
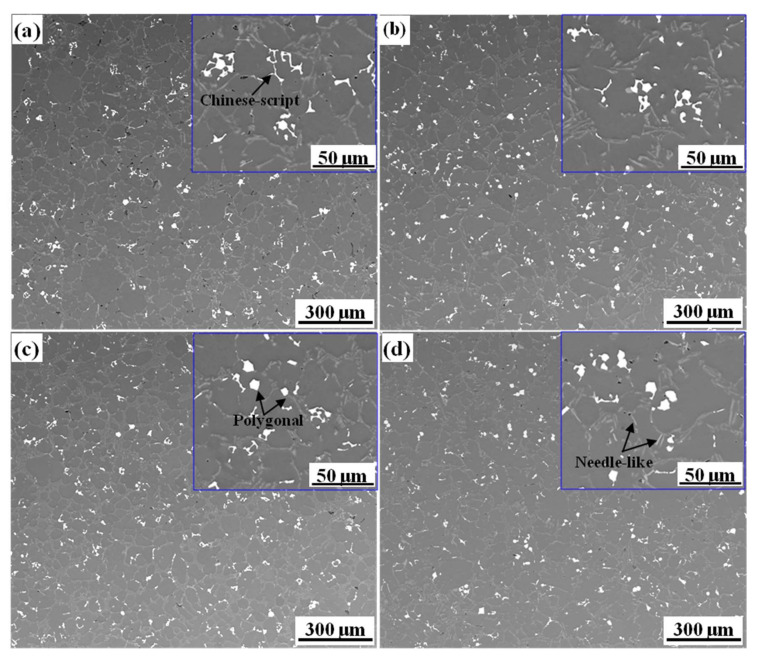
The morphology of the Fe-rich phase of the alloy with different initial Fe content: (**a**) 0.8%; (**b**) 1.2%; (**c**) 1.8%; (**d**) 2.4%.

Figure 2 shows the SEM images of the Fe-rich phase in the alloy with different initial Fe contents. After adding Mn and melt treatment, the morphology of the Fe-rich phase mainly consisted of Chinese-script, polygons and plate-like morphologies. Figure 2a shows the SEM images of the Fe08 alloy, which was dominated by coarse Chinese-script, as well as a bit of polygonal shape. With the initial Fe content increase, the number of polygonal phases increased significantly, while the number of Chinese-script phases reduced, which was accompanied by a reduction in size and an increase in the compactness. It is worth noting that slight plate-like phases appeared in Fe24 alloy. As shown in Table 2, three typical morphologies Fe-rich phases are composed of Al, Si, Mn, and Fe. However, the Mn content in the polygonal phase was significantly higher than the other two Fe-rich phases. The Mn content in plate-like phases was much lower than the other two types of Fe-rich phases. Based on the morphology and chemical composition of the Fe-rich phase, the polygonal and Chinese-script phases were identified as α-Fe, and the plate-like phase was β-Fe [27]. Therefore, with the increase of Fe content, the type of Fe-rich phase transformed a single α-Fe into a mixed type with α-Fe and β-Fe. The morphological evolution process is: coarse Chinese-script + polygon → polygon + fine and compact Chinese-script → polygon + fine and compact Chinese-script + plate-like.

**Figure 3:**
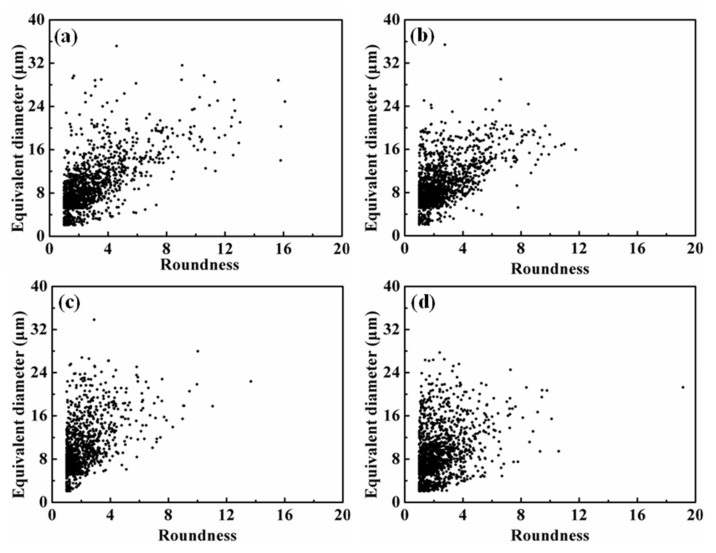
The relationship between the equivalent diameter of the iron-rich phase and its roundness with different Fe content: (**a**) 0.8%; (**b**) 1.2%; (**c**) 1.8%; (**d**) 2.4%.

Table 3 shows the image statistics of the Fe-rich phase using image analysis software. After Fe removal by gravity settlement, the area fraction of the Fe-rich phase in the ingot dropped to 2.04–2.61%, which was more than 55% lower than that of in Al-7Si-1.0Fe-1.2Mn [17]. With the increase of the initial Fe content, no obvious tendency was found in the area fraction change, but the area fraction was basically consistent with the total content of residual Mn and Fe, as listed in Table 1. Figure 3b demonstrates that the Fe content has no significant effect on the average size of the Fe-rich phase, but the equivalent diameter of the top 10 particles decreased first and then increases. In particular, the equivalent diameter of the top 10 particles in Fe18 alloy was 17.78% lower than that of Fe08 alloy. Figure 3c presents that the roundness value of Fe08 alloy was larger than that of other alloys, which indicated that the increase of initial Fe content promoted the compactness of the Fe-rich phase. In addition, the variation trend of the roundness of the top 10 particles was consistent with the average value.

**Figure 4:**
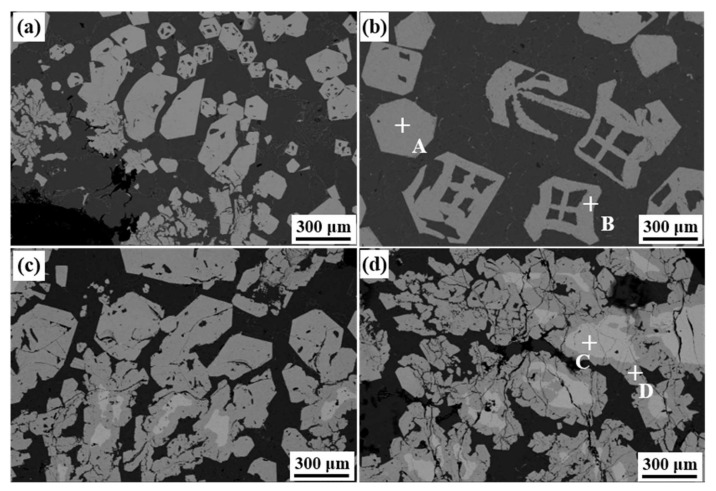
Microscopic morphology of slag with different Fe content.(**a**) 0.8%; (**b**) 1.2%; (**c**) 1.8%; (**d**) 2.4%.

Figure 3 shows the relationship between the equivalent diameter and the roundness of the Fe-rich phase in alloys with different initial Fe content. The equivalent diameter of the Fe-rich phase was mainly distributed between 5 and 15 μm, while the roundness was main in the range of 1–5. Figure 4a shows that the equivalent diameter and roundness ranges of the Fe-rich phase in Fe08 alloy were 2–35 μm and 1–16 μm, respectively. With the increase of Fe content, the equivalent diameter and roundness range was significantly reduced, where the equivalent diameter was narrowed to 2–28 μm, and the roundness was 1–12 μm.

Figure 4 shows the SEM images of sludges settled on the bottom of the furnace. The morphology of the Fe-rich phase in the slag was mainly divided into three types: polygon, hollow polygon and irregular-shape, and its size ranged from 30 μm to several millimeters. Figure 4a,b present that the morphology of the Fe-rich phase in Fe08 and Fe12 slag was mainly solid polygons and hollow polygons, and the corresponding equivalent diameter did not exceed 800 μm. Figure 4c,d shows that the morphology of the Fe-rich phase gradually transformed into an irregular-shape with the increase of Fe content. Meanwhile, the number and size of irregular-shape gradually increased, and their maximum size exceeded 3 mm. Note, that an obvious interface divided the irregular-shape phase into two layers. Table 4 shows that the chemical composition of the outer layer in the irregular-shape phase, polygon and hollow polygon was similar, while the Mn content of the inner layer in the irregular-shape phase was higher than the above-mentioned Fe-rich phase, and the corresponding Fe content significantly reduced.

**Figure 5:**
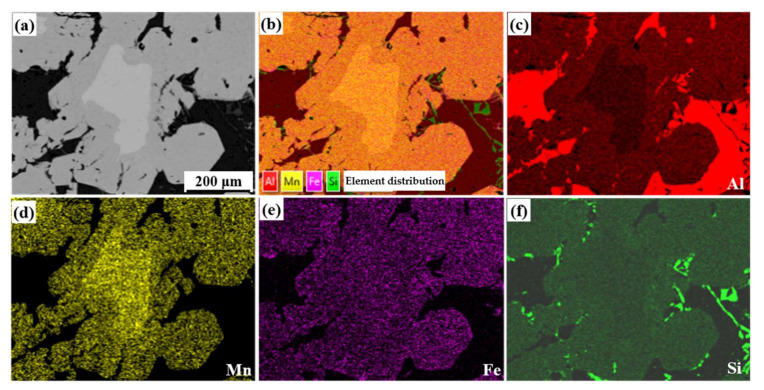
Elements mapping of typical Fe-rich phase in slag. (**a**) SEM image; (**b**) Comprehensive distribution of elements; (**c**) Al; (**d**) Mn; (**e**) Fe; (**f**)Si.

Figure 5 shows the composition mapping of the irregular-shape phase, and the Mn content in the inner layer was significantly higher than that in the outer layer. Our previous research [19] found that the irregular-shape phase with double-layer structure was peritectic, both of the inner and outer layers belonged to α-Fe, but the low diffusion coefficient of Fe and Mn in the solid phase hindered their migration during the peritectic reaction, leading to obvious delamination of peritectic structure.

### 3.3. The Effect of Initial Fe on Tensile Properties

**Figure 6:**
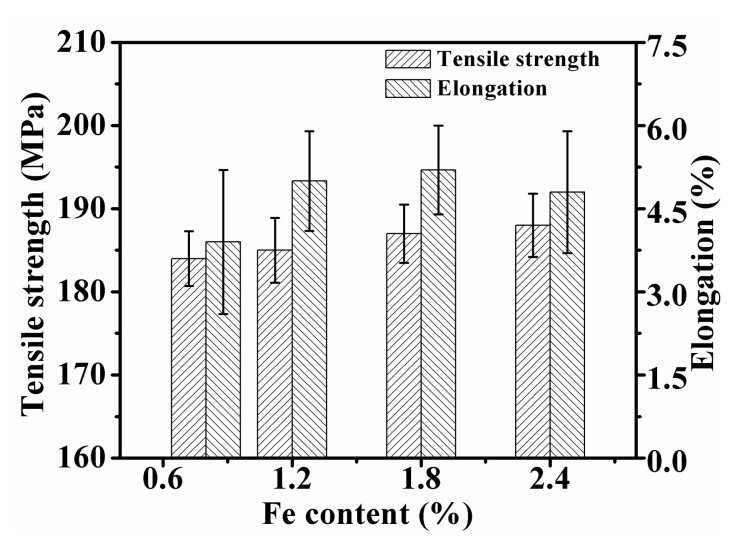
Effect of Mn/Fe ratio on tensile mechanical properties of alloy.

Figure 6 shows the as-cast tensile mechanical properties of alloys with different initial Fe content. After adding Mn and melt holding treatment, the tensile strength of the alloys stayed at 185 ± 5 MPa, and the elongation was 3.5~5.2%, which was similar to the as-cast properties of the unmodified A356 aluminum alloy [28]. With the increase of Fe content, the elongation presents a trend of first increasing and then decreasing, where the maximum elongation appeared in Fe18 alloy, which was 48.6% higher than that of Fe08 alloy.

**Figure 7:**
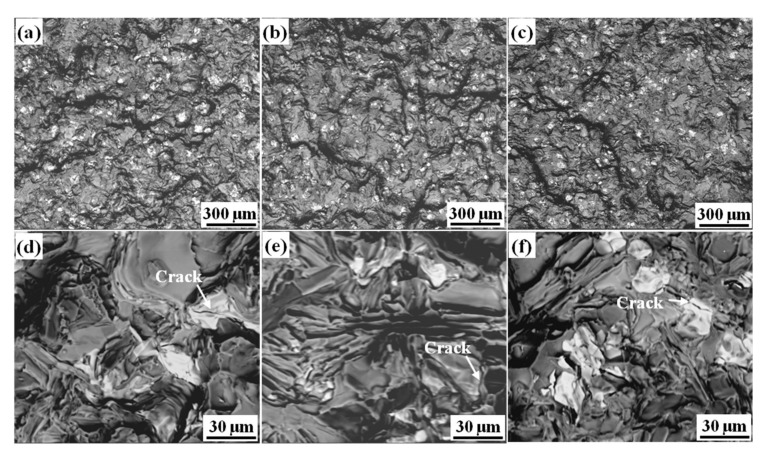
Fracture morphology of alloys under different Fe content: (**a**,**d**) 0.8%; (**b**,**e**) 1.8%; (**c**,**f**) 2.4%.

Figure 7 shows the fracture morphology of the alloys with different initial Fe content. The gray-white area represented the Fe-rich phase, and the gray-black area showed the matrix and Si phase. Figure 7a,d shows a certain amount of Fe-rich phase (size ≥ 30 μm) with a relatively smooth surface on the fracture surface of Fe08 ally. Meanwhile, some secondary cracks were found on the surface of the Fe-rich phase, which indicated that the crack initiation was easier to initiate in the coarse Fe-rich phase. With the increase of Fe contents, the number of large-size Fe-rich phases was obviously decreased, and the uniformity of distribution of the Fe-rich phase was improved. Secondary cracks were found both on the surface of the Fe-rich phase and eutectic silicon, which indicated that the cracking tendency of these two phases was similar, as shown in Figure 7b,c,e,f.

## 4. Analysis and Discussion

The formation and settlement of the primary α-Fe is the main reason for Fe removal in the alloy. In order to optimize the settlement processes, scholars have been summarized empirical formulas for the formation tendency (sludge factor, SF) and temperature (T) of the primary α-Fe based on a large number of experiments, which are as follows [18,29]:

SF = 1 × w (Fe) + 2 × w (Mn) + 3 × w (Cr)(1)

T = 645.7 + 34.2 × [wt.%Fe]^2^ (2)

Formula (1) shows that the increase of Fe, Mn, and Cr content is beneficial to promote the formation tendency, Formula (2) indicates that the formation temperature is proportional to the square of the Fe content. In addition, many researchers found that the formation temperature of the primary Fe-rich was significantly impacted by the Mn content and cooling rate [12,19], which increased with the increase of the Mn content [17,19].

**Figure 8:**
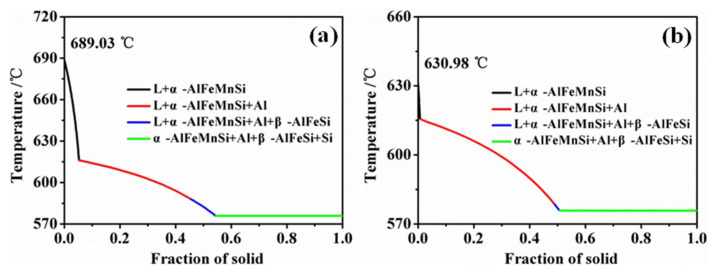
Equilibrium solidification curve of Fe18 alloy calculated by PANDAT^TM^. (**a**) Before melt treatment; (**b**) After melt treatment.

According to formulas (1), the initial SF value of the four test alloys reaches 2.72 and above, far exceeding the 1.30 required for the formation of the primary phase. According to formulas (2), the corresponding formation temperature is up to 667.58 °C and above, far exceeding the α-Al dendrite. Therefore, the chemical composition of the four test alloys provides the thermodynamic conditions for the formation and settlement of the primary phase. The formation temperature of the primary Fe-rich phase in Fe12 is ~689.03 °C (Figure 8a), which is close to the 694.95 °C calculated by formula (2). Preliminary studies have found that the Al-7.0Si-2.4Fe alloy preferentially formed a band-shape (irregular-shape) with a double-layer peritectic structure when the Mn/Fe ratio exceeds 0.7. The inner layer phase greatly consumed the Mn rather than equal amounts of Mn and Fe, which resulted in the Mn content used forming the outer layer. Moreover, other polyhedral primary phases had not increased at a higher Mn content, hence the Fe removal had no significant change. Therefore, the double-layer phase in Fe18 and Fe24 alloys reduced the effective content of Mn used for removing Fe. In addition, high initial concentrations of Fe and Mn cause greater component undercooling, which increases the driving force for the nucleation and growth of the Fe-rich phase. As a result, the primary Fe-rich phase with irregular shape and hollow morphology was formed. With the Mn consuming the peritectic and polygonal phase transformation instead of the inner layer phase transition. The different types of primary phases contacted and collided during the settlement process, which piled together and increased the equivalent diameter to several millimeters.

**Figure 9:**
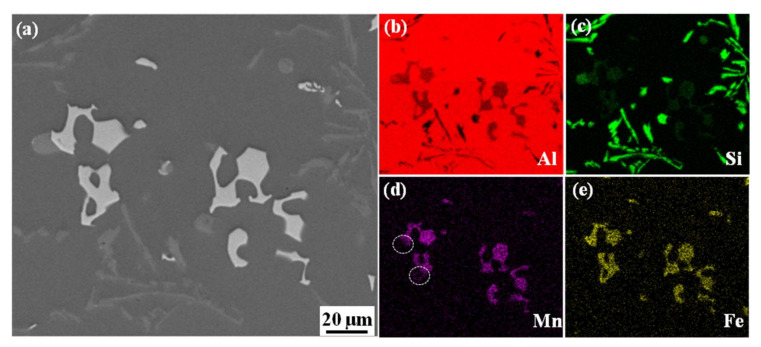
SEM image and composition distribution of Fe-rich phase with typical morphology: (**a**) SEM; (**b**) Al; (**c**) Si; (**d**) Mn; (**e**) Fe.

As the Mn further consumed, the SF value in residual melt decreased to the critical value, and the primary Fe-rich phase formed at this time showed a characteristic of regular shape and small size due to the low growth driver and were difficult to settle to the bottom of the furnace within the holding time [30]. Figure 8b shows the formation temperature of the primary Fe-rich phase was about 630.98 °C in the residual melt of Fe12, which was slightly higher than that of α-Al dendrites (615.16 °C). Therefore, the temperature difference between the primary Fe-rich phase and α-Al dendrites provided thermodynamic conditions for forming small size and regular primary phases. In the subsequent pouring process, the above fine particles acted as nucleation sites and promoted a coupling structure composed of polygonal and Chinese script-like. The Mn and Fe far from the nucleation sites nucleated on the surface of the oxide film, and finally form a Chinese-script phase. Figure 9 shows the Mn concentration in the Chinese-script area was significantly lower than that of the polygon in the same coupling structure, which indicated that the formation of the Chinese-script phase was later than that of the polygonal phase. Therefore, the increase of the initial Fe content promoted the consumption of Mn in the double-layer phase, resulting in an increase in the Fe content and a decrease in Mn content.

The Fe-rich phase is a typical hard phase in aluminum alloys and causes stress concentration and cracking, especially the large-size and plate-like morphology [31,32,33]. Therefore, with the increase of Fe content, the morphology of the Fe-rich phase changed from a large-size Chinese script to a denser Chinese script and polygonal, which was beneficial to improve the plasticity of the alloy. However, the presence of a plate-like phase may hinder the further improvement of the plasticity of high-Fe-content alloys.

## 5. Conclusions

(1) With the increase of the initial Fe content, the Fe content of the alloys gradually increased, while the corresponding Fe removal efficiency gradually increased to 77.67%.
(2) With the increase of the initial Fe content, the Fe-rich phase type in the alloy changed from a single type of α-Fe to a mixed type of α-Fe and β-Fe. The morphological evolution of the Fe-rich phase was listed as follows: coarse Chinese-script + polygon → dense Chinese-script + polygon → polygonal + dense Chinese-script + plate-like.
(3) With the increase of the initial Fe content, the morphology of the Fe-rich phase in the slag changed from a polygonal shape to an irregular-shape with a two-layer structure. The formation and increase of the inner layer with high Mn content in the irregular-shape phase was the main reason for the increase of Fe content in the alloy.
(4) With the increase of the initial Fe content, the plasticity of the alloy increased obviously, but the formation of the β-Al_5_FeSi with plate-like morphology in higher Fe content alloy might hinder the further improvement of the plasticity.

## Figures and Tables

**Table 1:** The chemical composition and Fe removal efficiency of the alloys.

NO.	Initial Composition				Final Composition				Fe Removal Efficiency %
	Si	Fe	Mn	Al	Si	Fe	Mn	Al	
Fe08	7.20	0.80	0.96	Bal.	7.29	0.44	0.48	Bal.	43.80
Fe12	7.19	1.20	1.44	Bal.	6.85	0.50	0.50	Bal.	58.30
Fe18	7.15	1.80	2.16	Bal.	6.90	0.53	0.41	Bal.	70.56
Fe24	7.21	2.40	2.88	Bal.	7.23	0.56	0.34	Bal.	77.67

**Table 2:** Chemical composition of Fe-rich phase in alloy (wt.%).

Test Point	Morphology	Al	Si	Mn	Fe	Identified Compounds
A	Polygonal	60.35	11.39	15.47	12.80	α-Fe
B	Chinese script	62.64	10.76	11.21	15.38	α-Fe
C	Plate-like	80.34	12.78	4.62	21.92	β-Fe

**Table 3:** The morphological characteristics of the Fe-rich phase in alloy.

Initial Fe Content (wt.%)	Area Fraction (%)	Average Qeuivalent Diameter (μm)		Average Roundness	
		All	Top 10	All	Top 10
0.8	2.04 ± 0.09	9.32 ± 3.21	30.10 ± 2.01	2.72 ± 1.63	6.79 ± 4.53
1.2	2.61 ± 0.10	10.2 ± 3.64	26.70 ± 1.95	2.06 ± 1.00	3.48 ± 2.13
1.8	2.22 ± 0.08	9.35 ± 2.99	24.65 ± 2.21	2.31 ± 1.25	4.92 ± 2.26
2.4	2.49 ± 0.11	10.2 ± 3.60	26.45 ± 0.66	2.13 ± 1.09	2.90 ± 1.60

**Table 4:** Chemical composition of Fe-rich phase in slag (wt.%).

Test Point	Morphology	Al	Si	Mn	Fe	Identified Compounds
A	Polygonal	56.98	11.26	19.84	11.93	α-Fe
B	Hollow polygonal	59.38	9.38	19.18	12.06	α-Fe
C	Inner	54.34	9.00	33.67	3.00	α-Fe
D	Outer	60.34	8.28	20.10	10.74	α-Fe

## Data Availability

The datasets generated and analyzed during the current study are available from the corresponding author on reasonable request.
